# The effect of reducing energy density, via the addition of water to dry diet, on body weight and activity in dogs

**DOI:** 10.1017/jns.2017.43

**Published:** 2017-08-29

**Authors:** Janet E. Alexander, Alison Colyer, Penelope J. Morris

**Affiliations:** WALTHAM Centre for Pet Nutrition, Waltham-on-the-Wolds, Melton Mowbray LE14 4RT, UK

**Keywords:** Dietary energy dilution, Body-weight management, Physical activity, Canine nutrition, MER, maintenance energy requirements, TMC, total moisture content

## Abstract

Approximately 40 % of pet dogs are estimated to be overweight and this is associated with health conditions significantly reducing life span and quality. In cats, dietary energy dilution has been reported to increase activity levels and aid healthy body-weight maintenance. Our aim was to investigate this in dogs. For 28 d, a complete and balanced standard dry diet, hydrated to a total moisture content (TMC) of 72 %, was offered to forty-six dogs at individual maintenance energy requirements (MER). Intake, body weight and activity were measured. For the following 28 d, the dry diet was offered at 200 % of MER with or without hydration (7·6 or 72 % TMC) and measurements repeated. When offered diets in excess, body weight increased significantly faster (19·3 g/d) with the hydrated diet (*P* = 0·001), but activity levels did not change from baseline (*P* = 0·392), while activity reduced significantly with the dry diet (about 15 %; *P* < 0·001). Dogs completely compensated for the reduction of dietary energy content, indicating that this is not a useful strategy for maintaining body weight when offered excess food.

It has been estimated that approximately 40 % of pet dogs are overweight and this is associated with health conditions that significantly reduce life span and quality^(^^1^^)^. It has been shown that obesity in pets is often due to too much food being offered^(^^2^^)^; strategies are therefore required to enable dogs to maintain a healthy body weight even when excess food is available. Dietary energy density dilution by the addition of water has been suggested as a strategy for weight management in humans^(^^3^^)^. This has also been suggested for cats as they have been reported not to regulate food intake if dietary energy density changes^(^^4^^–^^9^^)^. When dietary energy density is reduced by the addition of moisture, cats do not increase their energy intake to fully compensate and therefore gain less body weight when offered hydrated diet in excess of energy requirements^(^^6^^–^^9^^)^. However, little is known about the effect of dietary energy dilution on energy regulation in the dog and it is possible that differences in digestive physiology and feeding behaviour could lead to differences between dogs and cats. Many dog breeds express a preference for large, infrequent meals, reflecting the competitive feeding behaviour of their ancestors, while cats often take several small meals, reflecting multiple kills of small prey in the wild^(^^10^^,^^11^^)^. The physiology of the dog and cat digestive systems parallel differences in feeding behaviour. Cats have smaller stomachs and relatively short small intestines consistent with frequent small meals^(^^12^^)^. Dogs have larger stomachs evolved to receive infrequent large meals and a longer small intestine which is required for complete absorption^(^^12^^)^.

A study to determine the effect of increasing dietary volume by incorporating air into dry expanded dog food reported that increasing dietary volume decreased energy intake^(^^13^^)^. The dilution of dietary energy density by the addition of fibre has also been suggested as a strategy to reduce energy intake in dogs, although such studies have produced conflicting results. In one study, obese dogs fed a high-fibre food were reported to reduce energy consumption, body weight and body fat compared with those fed a low-fibre food^(^^14^^)^. In contrast, the inclusion of soya hull fibre into extruded diet did not reduce the food intake of beagle dogs fed amounts that exceeded their energy requirements^(^^15^^)^. To our knowledge, only one previous study has specifically examined the effect of increasing dietary moisture levels on energy intake in the dog. Janowitz & Grossman^(^^16^^)^ added cooked meat juice and/or cellulose to a dried commercial dog food to dilute the energy content. The results suggested that dogs could compensate for dietary energy dilution by increasing intake; however, marked individual differences were observed in the rate and precision with which energy adjustments were made. The aim of the present study was therefore to determine the effect of increasing levels of dietary moisture by the addition of water to dry diet, on intake, body weight and activity in dogs offered energy in excess of requirements.

## Experimental methods

This work was approved by the WALTHAM Animal Welfare and Ethical Review Body and followed UK Home Office Code of Practice guidelines for animal welfare.

### Animals

A total of forty-six neutered miniature schnauzers aged 2–7 years were pair housed, but fed individually at the WALTHAM Centre for Pet Nutrition. All dogs were within 5 % of their veterinarian-determined ideal body weight at the start of the study and deemed clinically healthy following examination. Exercise and socialisation sessions were standardised, and *ad libitum* drinking was available water throughout. Dogs were paired for age and sex and randomised to one of two dietary treatment groups.

### Study design

For a 28 d baseline period all dogs were offered their maintenance energy requirements (MER) of a single batch of complete and balanced dry diet hydrated with water to a total moisture content (TMC) of 72 %. Over the following 28 d, dogs were offered 200 % individual MER daily of either the dry diet (7·6 % TMC) or the hydrated dry diet (72 % TMC). In accordance with routine feeding conditions, two meals per d were presented for 30 min during which evaporation did not exceed 2 % of the total weight of the diluted diet (data not shown).

### Measures

Daily dietary intake (g) was recorded individually within 10 min of the end of feeding. Percentage consumed was calculated on a per d basis. Body weight (kg) was recorded twice weekly in the fasted state (>5 h). Activity levels were determined in three uninterrupted 72 h sessions in week 3 of phase 1 and weeks 1 and 3 of phase 2 using Actical™ accelerometers.

### Statistical analysis

The study had 80 % power to detect a between-group body-weight difference of 1·25 % per week over 4 weeks. Five dogs (one dry diet and four hydrated diet) were removed early due to body-weight gain above the pre-set welfare limit of +20 % of ideal. Removal was informative to body weight as primary endpoint, therefore to enable a calculation of rate of change, a linear regression was performed from day 3 of phase 2 onwards for each dog. Rate of body-weight change was analysed by ANCOVA, with baseline body weight as a covariate. Intake was analysed by mixed-model analysis (MMA). For dogs removed from the study, 5–7 values were missing. All but one of the previous intakes for these dogs were 100 % of offered, therefore this was imputed for the missing data. Activity data were taken before removal and were analysed by MMA; baseline activity was a covariate. The Bonferonni-corrected test level, for three endpoints, was 0·0167 (0·05/3).

## Results

**Fig. 1.**
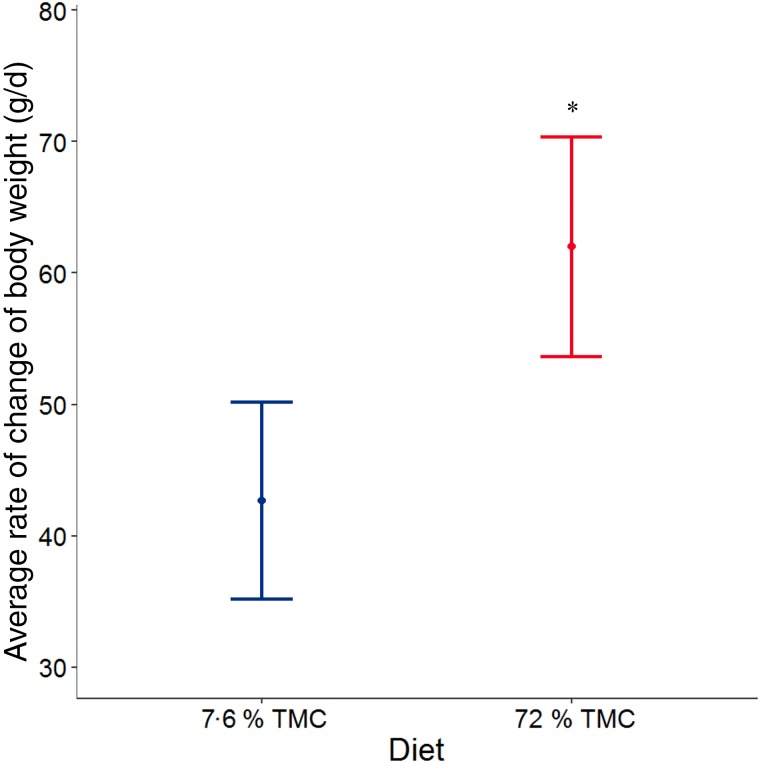
Average rate of change of body weight (g/d), adjusted for baseline body weight by diet (*n*   23 per diet group). Values are means, with 95 % confidence intervals represented by vertical bars. * Mean value was significantly different from that for 7·6 % total moisture content (TMC) (*P* = 0·001).

When offered 200 % of MER of the hydrated diet, dogs gained body weight significantly faster than those offered dry diet (*P* = 0·001), by 19·3 (95 % CI 8·1, 30·5) g/d (Fig. 1).

### Intake

Mean percentage intake reduced over the excess feeding phase. In the first 7 d, dogs offered the dry and hydrated diets consumed 98·3 and 97·2 %, respectively and 84·3 and 86·9 % in the last 7 d. Mean percentage consumed was not significantly different between groups at any time over the 4 weeks when 200 % MER was offered (*P* > 0·017); data not shown.

### Activity

There was no between group difference in total activity units/d during the baseline phase (*P* = 0·599) or the first (*P* = 1·000) or third week (*P* = 0·134) of the excess feeding phase (Table 1). When mean activity levels in weeks 1 and 3 of the excess feeding phase were compared, dogs offered the 7·6 % TMC diet were reduced (*P* < 0·001) by 42 267 units (20 532, 64 002) (Table 1). In contrast, the activity levels of dogs offered the 72 % TMC diet did not reduce (*P* = 0·392) over this period (Table 1); however, the changes in activity between the first and third weeks were not significantly different between the two diet groups (*P* = 0·088).

**Table 1.** Average activity (total units/d), adjusted for baseline average activity by diet (7·6 % total moisture content (TMC) *n* 23, 72 % TMC *n* 22) (Mean values and 95 % family-wise confidence intervals)

	Activity (total units/d)				
	Week 1		Week 3		
Diet TMC (%)	Mean	95 % CI	Mean	95 % CI	P*
7·6	274 543·1	250 377·1, 298 709·0	232 276·1	208 110·2, 256 442·1	<0·001
72	275 452·3	250 355·5, 300 549·1	261 751·2	237 035·6, 286 466·8	0·3917
*P*†	0·9999		0·1336		

* *P* value for within-diet difference between weeks.

† *P* value for between-diet difference within weeks.

## Discussion

Our data indicate that dogs compensate for dietary energy dilution through the addition of water to a dry diet. Body-weight increase was observed to be more rapid when a hydrated diet rather than a dry diet was offered in excess of energy requirements. Therefore this is not a useful strategy for weight management in an overfeeding environment.

Many species, including humans, compensate for dietary energy dilution by increasing their intake^(^^17^^–^^19^^)^. In contrast, a number of studies^(^^4^^–^^9^^)^ have demonstrated that cats do not quickly increase their energy intake to fully compensate for energy dilution and this strategy has been suggested to aid healthy body-weight maintenance when overfeeding occurs. However, the data presented here indicate that dogs do compensate for changes in dietary energy content, at least up to the 100 % excess provided in this study. Despite the increased volume, dogs offered the hydrated diet consumed the same percentage of the ration and therefore the same percentage of MER as those offered the dry diet. This dissimilarity to cats may be because dogs are ‘adaptive’ rather than obligate carnivores and natural scavenging behaviour in the wild means the nutritional composition of their diet can vary more widely than cats^(^^11^^)^, requiring the ability to adapt to variations in dietary energy content. Furthermore, the digestive systems of dogs and cats may handle particles and fluids differently; in the fed state, the cat stomach retains smaller particles than in dogs^(^^12^^,^^20^^)^, possibly influencing the comparative satiating effects of dry and hydrated diets.

Although there was no significant difference in intake, the rate of body-weight change was significantly greater for dogs offered the hydrated diet. A number of factors could explain this, for example, it is possible that the hydrated diet had greater digestive efficiency than the dry diet, thus suppling more energy. Water availability is one factor that determines the rate of starch digestibility and therefore energy availability^(^^21^^)^. Here, no attempt was made to calculate the digestive efficiency of the diets. However, Cameron *et al*.^(^^7^^)^ reported no difference in the apparent energy absorption efficiency and net energy assimilation of a dry diet hydrated by the addition of water to 40 % total moisture. Changes in body composition could also account for the difference in body-weight gain. If dogs receiving the hydrated diet maintained or gained more lean body mass than those receiving the dry diet, a difference in body weight might have been expected. Body composition analysis was not carried out in this study, but should be further investigated.

Physical activity levels were significantly lower at the end of the excess feeding phase in the dry diet group. This could not be solely due to weight gain as this did not occur when the hydrated diet was fed even though the change in body-weight gain was greater. Neither could this be explained by having greater opportunity to exercise than those receiving the dry diet as exercise regimens were highly regulated and groups randomised across three kennels. It is possible that the change in activity levels was linked to hydration level. It has been demonstrated that the total moisture intake of dogs is significantly reduced when fed a low-moisture (7 % TMC) diet and this could lead to reduced hydration^(^^22^^)^. In humans, dehydration by as little as 1 % loss of body mass results in reduced physical performance and fatigue^(^^23^^)^. Further research is needed to address the effect of hydration status on physical activity in dogs. One limitation of the present study was that water consumption, and urine and faecal volume were not measured, and thus hydration levels not estimated.

The hypothesis that lowering the energy density of the diet via the addition of water to dry food would result in a reduction in body-weight gain in dogs offered diets in excess of energy requirements was not proven.
